# Community health worker and family support for addiction treatment services

**DOI:** 10.1186/s13011-026-00702-z

**Published:** 2026-01-22

**Authors:** Li Li, Tuan Anh Nguyen, Hieu Ngoc Nguyen, Van Thi Nguyen, Ha Thi Thanh Nguyen, Thang Hong Pham

**Affiliations:** 1 Department of Psychiatry and Biobehavioral Sciences, David Geffen School of Medicine, University of California, Los Angeles, 760 Westwood Plaza, Los Angeles, CA 90024 USA; 2 National Institute of Hygiene and Epidemiology, 1 Yersin Street, Hanoi, 100000 Vietnam; 3 Hanoi Medical University, 1 Ton That Tung Street, Hanoi, 100000 Vietnam

**Keywords:** Community health workers, Family, Care support, People who use drugs, Vietnam

## Abstract

**Background:**

A growing body of research underscores the importance of incorporating family support and community health worker engagement into community-based addiction care. This study developed and applied context-specific indicators tailored to the distinct roles of family members and CHWs in addiction services.

**Methods:**

This study utilized baseline data from an ongoing randomized controlled trial conducted in 2024 across 58 communities in Vietnam. Participants included 174 community health workers and 690 family members of people who use drugs. Context-specific support indicators were developed separately for CHWs and family members. Multiple regression analyses were used to identify individual, familial, and work-related factors associated with support for addiction services.

**Results:**

Both the community health worker support and family member support scales demonstrated acceptable reliability. Among community health workers, higher job satisfaction and greater confidence in delivering addiction services were significantly associated with stronger support for addiction care. Among family members, higher levels of family functioning and coping were positively associated with support for addiction services.

**Conclusion:**

Findings highlight the potential of enhancing community health workers’ confidence and job satisfaction, as well as promoting family functioning and coping, as actionable strategies to strengthen addiction service systems and inform future interventions. By utilizing context-specific indicators, this study provides a more nuanced understanding of support mechanisms within community-based addiction care.

## Introduction

Globally, a growing body of research highlights the importance of integrating family support and community health worker (CHW) involvement into addiction care in community settings [1, 2]. Such community-based support systems are particularly effective because they offer multiple layers of accessible and sustainable assistance and resources [3–5]. Prior studies have shown that family members of people who use drugs (PWUD) often serve as primary caregivers and advocates, providing both emotional and logistical support to individuals undergoing addiction treatment. Their involvement has been associated with improved treatment engagement and better outcomes [6–9]. Similarly, CHWs play a critical role in supporting addiction services through education programs, harm reduction strategies, and case management, helping ensure that PWUD receive continuous care beyond clinical settings [10–12].

Incorporating family and CHW support into addiction treatment services presents both opportunities and challenges. In Vietnam, most PWUD live with their families, and family presents the principal source of financial support and care [13, 14]. While supporting their loved one’s care and treatment, family members often experience significant emotional, social, and financial burdens [15, 16]. Vietnam has an existing commune health system that forms the grassroots public health network to provide essential preventive and treatment services in the community. However, funding and infrastructure for addiction treatment at the commune level remain minimal [17] and CHWs face high workloads and inadequate training to meet the complex needs of patients [18]. In recent years, Vietnamese government has made notable progress in promoting harm reduction initiatives and community-based treatment models [19, 20]. However, gaps remain in systematically including families and CHWs in addiction service delivery [21–23]. Understanding the nature of their support and the factors associated with it is essential for addressing these gaps.

This study explores support in addiction services by CHWs and family members in Vietnam. Unlike most existing studies, including our own prior work, which have relied on general measures of social support, this study developed and utilized context-specific indicators tailored to the unique roles of family members and CHWs in addiction services. By addressing this methodological gap, the findings of this study can offer a more precise understanding of support mechanisms and inform future interventions aimed at enhancing the involvement of CHWs and families, ultimately strengthening community support networks across the addiction care continuum.

## Methods

### Participants

This study utilized baseline data from an ongoing randomized controlled trial conducted in three Vietnamese provinces—Ninh Binh, Da Nang, and Can Tho [24]. Between May and July 2024, assessments were carried out in 58 communities across these provinces. Participants received an incentive of 230,000 VND (approximately $10 USD) for completing the assessment. The study protocol and data collection materials were approved by the Institutional Review Boards (IRBs) of collaborating institutions in both the United States and Vietnam.

In Vietnam, commune health centers typically employ three to five community health workers (CHWs), including doctors, assistant doctors, nurses, midwives, and pharmacists. CHWs were eligible to participate if they were at least 18 years old and actively engaged in delivering health services to residents. Support staff such as cleaners, accountants, and security personnel were excluded from the study. During recruitment, project staff provided detailed information about the study’s objectives, procedures, and potential risks and benefits. Participation was emphasized as voluntary and unrelated to employment responsibilities. CHWs were assured that their decision would not impact their job status. Written informed consent was obtained before data collection. A total of 174 CHWs were enrolled and included in this study.

Family member recruitment began with nominations from PWUD participants who had consented to participate in the study. Each PWUD was asked to nominate a household family member who provided care or support. The research team then conducted confidential meetings with the nominated individuals. Eligibility criteria for family members included: (1) being 18 years of age or older; (2) being an immediate or extended family member of the participating PWUD; and (3) being aware of the PWUD’s substance use. All family participants provided written informed consent prior to assessment. A total of 690 family members were enrolled, with a refusal rate of 12%.

### Data collection

The CHW assessments were conducted individually in private offices at each commune health center and administered in Vietnamese. Surveys were completed using Audio Computer-Assisted Self-Interviewing (ACASI), in which questions were presented both audibly via headphones and visually on a laptop screen. Participants entered their responses directly into the computer. On average, the assessment took approximately 30 min to complete. A trained interviewer was present to provide clarification regarding survey items or technical issues related to ACASI, if needed.

Family member participants completed a structured survey in private settings, either at local health centers or an alternative location of their choosing, to ensure comfort and confidentiality. Assessments were administered in a one-on-one, face-to-face format using the Computer-Assisted Personal Interviewing (CAPI) method. Trained interviewers read the survey questions in Vietnamese and entered responses directly into laptop computers. Each session lasted approximately 45 to 60 min.

### Measures

Support for addiction services was measured separately for CHWs and family members. The items included in each support scale are listed in Table 1. These items were developed based on findings from prior projects and the formative phases of the current study. For each item, participants reported how frequently they had made efforts to support PWUD in the past three months. Each scale included eight items, and the total score was calculated as the sum of item responses. Higher scores reflected greater levels of support for addiction services. The CHW support scale ranged from 8 to 40 (Cronbach’s α = 0.97), and the family member support scale also ranged from 8 to 40 (Cronbach’s α = 0.91).

**Table 1:** Questions in support for addiction services scales and item-to-total correlation

Item-to-Total Correlation	
*Community Health Workers* (*n* = 174)	
1. How often did you provide counseling to PWUD patients?	0.89
2. How often did you answer PWUD patients’ questions?	0.88
3. How often did you motivate PWUD to seek addiction treatment?	0.93
4. How often did you encourage PWUD patients to stay in addiction treatment?	0.94
5. How often did you ask about the reason if PWUD patients missed an appointment?	0.89
6. How often did you remind PWUD patients to have tests for prevention and treatment?	0.93
7. How often did you talk with PWUD about the harm of untreated addiction/drug use?	0.94
8. How often did you praise PWUD when making progress on seek/stay in treatment?	0.92
*Family Members* (*n* = 690)	
1. How often did you motivate [PWUD] to seek addiction treatment?	0.78
2. How often did you encourage [PWUD] to stay in addiction treatment?	0.71
3. How often did you accompany [PWUD] to addiction services?	0.69
4. How often did you suggest [PWUD] to talk to providers about addiction treatment?	0.79
5. How often did you talk with [PWUD] about impact of untreated addiction in family?	0.81
6. How often did you praise [PWUD] when he/she seek professional help for addiction?	0.84
7. How often did you try to keep [PWUD] away from dropping out addiction services?	0.83
8. How often did you remind [PWUD] about seeking/staying in addiction treatment?	0.81

Item-to-total correlation: Pearson correlation between an individual item and total scale score

Response categories: 1 = None of the time, 2 = A little of the time, 3 = Some of the time, 4 = Most of the time, 5 = All the time

Additional variables were collected from CHWs and included in the analysis. *CHW job satisfaction* was assessed using a four-item scale adapted from Karaferis et al. [25]. Items included: “You like doing the things you do at work,” “You feel a sense of pride in doing your job,” “Your job is enjoyable,” and “You feel that your job is meaningful.” Responses were rated on a five-point Likert scale from 1 (“Strongly agree”) to 5 (“Strongly disagree”). Items were reverse-coded, and a composite score was computed, with higher values indicating greater job satisfaction (range: 4 to 20; Cronbach’s α = 0.93). *CHW confidence in providing addiction services* was measured using a 13-item scale developed by the study team, based on a short version in Vietnam [26]. Participants rated their confidence in delivering addiction-related services across domains such as providing consultation, assessing service needs, engaging PWUD in treatment, supporting adherence, and offering long-term care. Each item was rated on a scale from 1 (“Not confident at all”) to 5 (“Very confident”). Higher total scores indicated greater confidence (range: 13 to 65; Cronbach’s α = 0.98). CHWs also provided demographic and professional background information, including gender, age, occupation, duration of employment at the current health center, and whether they had ever received training in addiction services.

For family members, demographic and background characteristics were collected, including gender, age, education level, self-reported financial status, and relationship to the PWUD. Relationships were categorized as spouse, parent, sibling, or other relatives (e.g., aunts, uncles, cousins). *Family functioning* was assessed using an adapted version of the Family Functioning Scale [27, 28]. Ten items were selected: five from family cohesion and five from sociability subscales. Participants rated how true each statement was for their family using a four-point Likert scale from 1 (“Very untrue”) to 4 (“Very true”). A higher summed score reflected better family functioning (range: 11 to 40; Cronbach’s α = 0.92). *Family coping* was measured with an adapted version of Namir et al.‘s coping scale [29], which assesses active cognitive and behavioral efforts to manage caregiving-related stress. This eight-item scale included statements such as “You have been taking action to try to make the situation better” and “You have been thinking hard about what steps to take to deal with the situation.” Responses were rated on a four-point Likert scale from 1 (“Not at all”) to 5 (“A lot”), with higher scores indicating greater coping capacity (range: 8 to 32; Cronbach’s α = 0.88).

### Data analysis

Descriptive statistics were used to summarize participant characteristics and support scale scores. Categorical variables were reported as frequencies (n) and percentages (%), while continuous variables were summarized using means and standard deviations (SD). We used t-tests and one-way analyses of variance (ANOVAs) to compare mean support scale scores across binary and categorical variables. In addition, we conducted correlation analyses to examine the relationships between continuous variables and the mean support scale scores. To investigate factors associated with support for addiction services, a series of multiple regression analyses was conducted. For CHWs, we estimated two hierarchical models. Model 1 included demographic characteristics, professional occupation, length of employment at the current health center, receipt of addiction service training, and job satisfaction. Model 2 additionally included confidence in providing addiction services. For family members, Model 1 included demographics, family situation, and family functioning, while Model 2 added coping with caregiving burden to assess its incremental contribution to family support for treatment. To assess the potential impact of multicollinearity, we examined the Variance Inflation Factor (VIF) for all independent variables included in the final models. The dependent variable in all models was the total score on the respective support for addiction services scale. Regression results are reported as estimated regression coefficients (β), standard errors (SE), and p-values. Statistical significance was determined at the α = 0.05 level.

## Results

Table 2 presents the characteristics of CHW and family member participants. For CHW the average age was 43 years, and approximately 72% were women. Among them, 20.7% were doctors, 38.5% assistant doctors, 19.5% nurses, 9.2% midwives, and 12% held other roles such as pharmacists. On average, CHW participants had worked at their health centers for 15.5 years, and 47% reported having received training related to addiction services. Bivariate analyses indicated that male gender, older age, and having received addiction service training were associated with higher levels of perceived support for addiction services among CHWs. Also shown in Table 2, most family members (77.5%) were female, and slightly more than half were aged 51 years or older. Participants had an average of 8.2 years of education, and 32.3% described their financial situation as poor. Regarding their relationship to the person who uses drugs (PWUD), 37.8% were spouses, 37.0% were parents, and the remainder were adult children, siblings, or other relatives. In bivariate comparisons, spouses reported higher levels of support for addiction services than parents or other relatives, and female family members reported higher support scores than male family members.

**Table 2:** Community health workers and family member participants’ characteristics

	*N*/Mean (SD)	%	Support Scale Mean (SD)	Pearson coefficient (*r*)	*p*
*Community Health Workers*	174		22.4 (8.6)		
Gender					
Male	48	27.6	25.7 (7.3)		< 0.001
Female	126	72.4	21.1 (8.8)		
Age	43.2 (8.6)			0.26	0.001
18 to 34	33	19.0	20.9 (9.0)		0.001
35 to 50	95	54.6	21.3 (8.7)		
51 and above	46	26.4	25.7 (7.4)		
Being a doctor					
Yes	36	20.7	24.0 (7.0)		0.20
No	138	79.3	22.0 (9.0)		
Years of working in health center	15.5 (9.8)			0.22	0.004
Received addiction training					
Yes	81	46.6	25.9 (7.6)		< 0.001
No	93	53.4	19.3 (8.3)		
Job satisfaction	18.1 (2.1)			0.22	< 0.001
Confidence in providing services	43.5 (12.1)			0.49	< 0.001
*Family Members*	690		28.3 (7.1)		
Gender					
Male	155	22.5	26.8 (6.2)		< 0.001
Female	535	77.5	28.7 (7.3)		
Age	52.1 (15.0)			-0.05	0.20
18 to 34	92	13.3	29.2 (7.2)		0.31
35 to 50	240	34.8	28.4 (7.4)		
51 and above	358	51.9	28.0 (6.8)		
Years of education	8.2 (3.9)			0.02	0.63
Reported poor financial status					
Yes	223	32.3	27.8 (6.9)		0.24
No	467	67.7	28.5 (7.1)		
Relationship with PWUD					
Spouse	261	37.8	29.7 (7.4)		< 0.001
Parents	255	37.0	28.0 (6.7)		
Siblings/Adult child/Others	174	25.2	26.4 (6.7)		
Family functioning	32.2 (5.5)			0.25	<0.001
Coping	24.3 (5.2)			0.34	< 0.001

Table 3 summarizes the results from multiple linear regression models examining factors associated with CHW support for addiction services. Age and years of service were included as continuous variables, while other characteristics were categorized for interpretability. In the adjusted model, age was no longer significantly associated with support scores. Being male and having received addiction service training remained significantly associated with higher levels of support. In addition, higher job satisfaction (standardized β = 0.153, *p* = 0.019) and greater confidence in providing addiction services (standardized β = 0.389, *p* < 0.001) were positively and significantly associated with CHW support for addiction services. Duration of employment at the health center and professional role were not significantly associated with support levels.

**Table 3:** Results from multiple regressions on CHW support for addiction treatment services

	Model 1		Model 2	
	β (SE)	*p*	β (SE)	*p*
*Community Health Workers*				
Being female	-0.230 (1.408)	0.002	-0.186 (1.290)	0.006
Age	0.091 (0.106)	0.386	-0.022 (0.099)	0.818
Years of working in health center	0.064 (0.095)	0.555	0.133 (0.087)	0.184
Being a doctor	0.011 (1.518)	0.903	0.038 (1.388)	0.552
Received addiction training	0.288 (1.273)	< 0.001	0.225 (1.175)	0.001
Job satisfaction	0.235 (0.279)	0.001	0.153 (0.260)	0.019
Confidence in providing services			0.389 (0.047)	< 0.001

β: *Standardized coefficient*; SE: *Standard error*

Table 4 presents factors associated with family member support for addiction services. Being a spouse (standardized β = 0.105, *p* = 0.021) was significantly associated with higher support scores. Other demographic characteristics, including gender, age, education level, and financial situation, were not significantly associated with support in the adjusted model. However, family functioning (standardized β = 0.142, *p* < 0.001) and coping (standardized β = 0.291, *p* < 0.001) were both positively and significantly associated with higher levels of family member support for addiction services.

**Table 4:** Results from multiple regressions on family member support for addiction services

	Model 1		Model 2	
	β (SE)	*p*	β (SE)	*p*
*Family Members*				
Being female	0.056 (0.687)	0.166	0.062 (0.659)	0.115
Age	0.031 (0.022)	0.499	0.017 (0.021)	0.698
Years of education	-0.006 (0.072)	0.890	-0.024 (0.069)	0.527
Poor financial status	0.005 (0.601)	0.891	-0.017 (0.578)	0.651
Being spouse	0.121 (0.685)	0.010	0.105 (0.658)	0.021
Family functioning	0.235 (0.049)	< 0.001	0.142 (0.049)	< 0.001
Coping			0.291 (0.051)	< 0.001

β: *Standardized coefficient;* SE: *Standard error*

## Discussion

Using context-specific support indicators, this study offers insights into the growing literature on community-based support for addiction services, focusing on two key groups: CHWs and family members. Differentiating support specific to addiction treatment services from general support for PWUD is essential, as context-specific indicators could enable a more precise and actionable understanding of the pathways through which CHWs and family members influence addiction service engagement and outcomes. The support indicators in this study were developed based on the lived experiences and behavioral roles of those supporting PWUD, allowing for the identification of support forms that are directly relevant to addiction treatment services. These context-specific indicators can potentially help to increase the practical utility of research findings for designing targeted interventions that align with the structural and relational dynamics of addiction care continuum at the family and community levels.

This study revealed significant associations between family member support and both family functioning and coping capacity, underscoring the role of the broader psychosocial and family environment in shaping a family member’s capacity and effort in supporting addiction treatment services. Supporters’ mental health is a key determinant of their effectiveness; psychological distress or caregiver burnout can impair their capacity to provide consistent and competent assistance [30, 31]. When family members of PWUD experience high caregiving burden or reside in dysfunctional family environments, their ability to offer support may be compromised due to a lack of energy, reduced focus, and diminished emotional clarity. Additionally, our findings indicate that being the spouse of a PWUD is significantly associated with higher levels of support. This aligns with prior research identifying spouses as primary caregivers who often shoulder a disproportionate burden of care, including facilitating access to health services [32, 33]. Particularly in Vietnam with a family-oriented culture, spousal caregivers often view caregiving as a natural part of their marital responsibilities [34]. Collectively, these study results highlight the need for family-based interventions that not only engage individual family caregivers but also aim to strengthen family dynamics and coping mechanisms.

The results of this study indicate that CHW support for addiction services is positively associated with their overall job satisfaction, prior training in addiction care, and confidence in service provision. It is not surprising to see job satisfaction and support correlated. On one hand, when CHWs can effectively assist patients in accessing addiction services, they may experience greater professional fulfillment and a stronger sense of efficacy, which can, in turn, enhance job satisfaction. On the other hand, as posited by self-determination theory, higher job satisfaction can foster intrinsic motivation [35]. Motivated CHWs are more likely to engage meaningfully with patients, persist in problem-solving efforts, and maintain long-term support. Similarly, CHWs who feel confident in their ability to deliver addiction-related services may be more proactive in advocating for treatment, offering accurate information and referrals, and navigating addiction care systems. Importantly, this study identified that both confidence and training in addiction care are key factors associated with CHW support, highlighting the importance of equipping CHWs with the knowledge and skills needed to engage PWUD effectively across the care continuum. When CHWs are trained with relevant models of addiction treatment and communication tools, they are more likely to value these resources [36], which could further strengthen their engagement in advocacy and support along the treatment continuum.

Several limitations should be acknowledged. First, the cross-sectional nature of the baseline data precludes causal inference. Second, all measures were based on self-report and may be subject to social desirability and recall biases, particularly given the sensitive nature of substance use and treatment-related experiences. Third, although the support scales were contextually grounded and demonstrated strong internal consistency, they were adapted for use in this study and would benefit from further psychometric evaluation, including assessments across diverse cultural and geographic settings. Despite these limitations, the study offers important empirical insights into community- and family-level dynamics that shape support for addiction treatment services in community-based settings.

This study has several implications for policy and practice. First, systematic integration of CHW-delivered support into addiction care could be accompanied by ongoing training to strengthen their confidence and competence in service delivery. Training programs may want to emphasize practical skill development, particularly in communication, counseling, and case management, through interactive sessions and supervised practice. In addition, policies that recognize CHW contributions and provide opportunities for professional growth within supportive work environments are essential to enhance job satisfaction, confidence, and long-term engagement in addiction care. Second, supporting family members, especially spouses, through stress management and coping interventions may enhance their sustained engagement in the addiction care process. Programs that offer psychoeducation on addiction, communication skills, and stress reduction techniques can strengthen families’ resilience and improve capacity to provide effective support in addiction care. In addition, integrating family or peer-support groups into community-based services may help reduce isolation and improve both family well-being and the continuity of addiction care. Finally, from a policy perspective, integrating CHW and family support into addiction care is important for strengthening Vietnam’s community-based care system. This approach aligns with Vietnam’s ongoing priorities for decentralized and people-centered care, underscoring the need for policy recognition, standardized training, and adequate resource allocation to ensure sustainability and equity in addiction service delivery.

## Conclusion

By examining two critical but often underexamined sources of support, CHWs and family members, this study contributes empirical evidence on factors associated with support for addiction treatment services in Vietnam. The findings suggest that higher CHW confidence and job satisfaction, as well as more positive family functioning and coping, are associated with stronger support for care in community settings. While causal relationships cannot be inferred, these results highlight potential leverage points for future intervention development and health system strengthening efforts. As Vietnam continues to expand the role of community-based services, integrating CHW capacity-building with family-centered approaches may be an important area for further intervention research.

## Data Availability

Data are available upon reasonable request after the completion of the trial.
